# Acute and chronic health risk assessment for inhalation and ingestion exposure in acrylic acid leak accidents

**DOI:** 10.1007/s11356-021-17293-w

**Published:** 2021-12-02

**Authors:** Hui-Been Lim, Si-Hyun Park, Hyong-Jin Hong, Ji-Yun Jeong, Hee-Seok Kim, Cheol-Min Lee

**Affiliations:** 1 Department of Nano & Biological Engineering, SeoKyeong University, Seoul, South Korea; 2 Department of Chemical & Biological Engineering, SeoKyeong University, Seoul, South Korea; 3 Environmental Health Center, SeoKyeong University, Seoul, South Korea

**Keywords:** Health risk assessment, Noncarcinogenic assessment, Inhalation exposure, Ingestion exposure, Acrylic acid, Chemical accident

## Abstract

We established a hypothetical acrylic acid leak accident scenario, conducted a health risk assessment of local residents, and compared an actual accident case to the hypothetical scenario. The exposed subjects were divided into four age groups, and a noncarcinogenic health risk assessment was conducted for inhalation and soil ingestion. In the hypothetical scenario, 40 tons of acrylic acid was leaked in Ulsan for 1 h from midnight on January 1, 2017. In the actual accident case, 3 L of acrylic acid was leaked in Hwaseong, Gyeonggi Province, for 1 h from 11:00 am on March 5, 2020. The environmental concentration of acrylic acid was calculated using the dynamic multimedia environmental model. Noncarcinogenic assessment of the hypothetical scenario showed the hazard quotient exceeded 1 across all age groups, suggesting that a health risk is likely to occur due to inhalation exposure to acrylic acid resulting from a chemical accident. In addition, Hazard_acute_ exceeded 1 until 2 h after the accident under the hypothetical scenario, indicating the likelihood of a health risk. Thus, we propose a methodology that can assess changing concentrations in a hazardous chemical leak from a chemical accident based on the time, place, the chemical’s behaviors in different environmental media, and the health risk posed by the exposure of the chemical to local residents in the area affected by the accident.

## Introduction

Rapid industrialization in recent years has resulted in the increasing use of chemicals in Korea and abroad, with more than 400 new chemical substances introduced annually. Chemical handling businesses in Korea are clustered in large industrial clusters (approximately 1000 in such areas as Sihwa, Yeosu, Ulsan, and Onsan) and in small and medium-sized industrial and agricultural clusters across the country (approximately 15,000). Therefore, the potential for a chemical accident exists anywhere in the country, and no one is safe from the risk of exposure (Kim et al. 2017). Consequently, it is necessary to review the response and post-accident management planning protecting people from chemical accidents.

The process industry, where advanced technology-intensive and complex processes are interconnected, encompasses the majority of chemical factories or chemical handling processes, and the consequences of any serious industrial accident (such as a fire, explosion, or leak) include casualties, property damage, environmental pollution, or harm to local residents (Lee, 2013). Leaks, in particular, can cause substantial social ripple effects. For example, gas or steam from a hazardous chemical leak can spread across the affected area and infiltrate the human body through the skin or respiratory system, causing lethal damage to local residents and workers (Jo et al. 2017).

Statistics from the National Institute of Chemical Safety (NICS) report that 110 chemical leaks have occurred as of November 2020. The most extensive chemical leak occurred in Nam-gu, Ulsan, at 2:00 pm on August 10, 2015. A total of 22 tons of hydrochloric acid leaked from a 40-ton tank in a workshop due to poor facility control (NICS, 2020).

There has been increasing concern about the risk of chemical hazards after large-scale chemical accidents with reported fatalities, including a hydrofluoric accident in Gumi. As a result, continuous studies on chemical accidents and national-level responses have been conducted. Under the Ministry of Environment (MoE), the NICS provides estimates of areas affected by chemical accidents in the Chemical Accident Response Information System (CARIS) and makes efforts to reduce damages arising from chemical accidents (Kim et al. 2020). Additionally, reports note that local residents lived within 1 km of some of the recent chemical accidents in Korea. These accidents posed a serious hazard to the residents, making it necessary to develop a method to predict and assess the impact of a chemical accident on the health of local residents (Park et al. 2020).

This study was conducted over 5 years between 2017 and 2021, as part of a study developing technology assessing the post-chemical accident human body impact and aiming to establish MoE response and post-accident management options for chemical accidents to protect local residents. We conducted a health risk assessment (HRA) on local residents affected by a hypothetical accident scenario to analyze inhalation and ingestion exposure for acrylic acid, applied the developed HRA method to an actual accident case, and compared the results obtained with those from the hypothetical scenario.

## Methods

### Chemical substance

In the current study, the target material selected for the HRA is acrylic acid, a substance requiring accident preparation under the Chemical Substances Control Act. Acrylic acid is an organic compound belonging to the class of unsaturated carboxylic acids. It is colorless, transparent at room temperature, and has a pungent odor similar to acetic acid. Due to its high solubility, it is easily soluble in various solvents such as water, alcohol, and benzene. In addition, it is very corrosive; thus, it can cause burns when exposed to steam and in contact with the skin. Synthetic resins made of acrylic acid are widely used to increase viscosity in lacquers, varnishes, and inks (Table 1).

### The dynamic multimedia environmental model

To calculate the concentration of acrylic acid remaining in the environment after an acrylic acid leak accident, we used the dynamic multimedia environmental model developed by Hong et al. (2020) in a study on developing technology to assess the post-chemical accident human body impact. A dynamic multimedia environmental model (DMEM) was established to identify the environmental behaviors of hazardous pollutants in the air, soil, and water, depending on the changing climate, by assuming abnormal conditions. This study reflected the movement of a hazardous pollutant via the air and established a model for soil and water, considering various conditions, including the impact of sedimentation (Park et al. 2020). The area for modeling was set as 15 km × 12 km, and the nested grid in the area was set as 0.1 km × 0.1 km. From the beginning of the accident to its end, meteorological information was collected from the Korea Meteorological Administration and used as the data required to operate the DMEM under the hypothetical scenario and the actual accident case.

### Exposure scenario

#### Hypothetical acrylic acid accident

To conduct an HRA of local residents affected by an acrylic acid leak accident, we referenced the largest ever leakage of chemicals in Korea, a 40-ton hydrochloric acid tank leak accident in 2015. We developed a hypothetical acrylic acid leak accident scenario. Ulsan, an area considered high-risk for a chemical accident due to its large industrial clusters, was the selected accident location under this hypothetical scenario. We assumed an hour-long 40-ton acrylic acid leak from a random factory at the center of Ulsan from midnight on January 1, 2017. The clearance time point after the accident was when the environmental concentration of acrylic acid was below 0.01 ppb. The acrylic acid endpoint concentration was assumed to be 0.01 ppb at the author’s discretion due to a lack of previous data.

**Table 1:** Virtual exposure scenario and actual accident used this study

Parameters	Scenario	
	Virtual scenario	Actual accident
Accident date	2017/01/01 00:00	2015/03/05 10:55
Accident point	Ulsan-Si	Hwasung-Si
Type	Leak	Leak
Amount	40 ton	3 L (3.153 kg)
Time	1 h	1 h
End point	21 days	1 days

#### Actual acrylic acid accident

An HRA was conducted for acrylic acid exposure in an actual accident case to compare the above method between the actual accident and hypothetical scenarios. The actual acrylic acid leak accident occurred at a factory in Hwaseong, Gyeonggi Province, on March 5, 2020, at 10:55 am. Similar to the hypothetical scenario, 3 L (3.153 kg) of acrylic acid leaked for 1 h. The end of the accident was assumed when the endpoint concentration of acrylic acid was achieved (below 0.01 ppb (25 °C, 1 atm), as in the hypothetical scenario (Table 2).

**Table 2:** Dose–response assessment data of acrylic acid used in this study

Classifications	Exposure pathway	Value	Toxicity	Reference
Cancer	Inhalation	N/A		
	Intake	N/A		
Non-cancer	Inhalation	RfC (mg/m^3^)	1 × 10^−3^	US EPA IRIS
	Intake	RfD (mg/kg/day)	5 × 10^−1^	

*RfC*, reference concentration factor; *RfD*, reference dose

### Health risk assessment

#### Chronic exposure

A chronic HRA was conducted based on a 4-step method suggested by the US National Research Council (NRC) and the US National Academy of Sciences (NAS) to reflect the characteristics of a chemical accident where the concentration of a hazardous chemical change over time (Park et al. 2020).

We found that acrylic acid can cause noncarcinogenic toxicity from inhalation exposure through the air and oral ingestion exposure through the soil. Dose–response information on noncarcinogenic toxicity, depending on the exposure pathway to determine the reference dose (RfD) and reference concentration (RfC), was obtained from the Integrated Risk Information System (IRIS) in the US Environmental Protection Agency (US EPA) (Table 3). The RfC value was used after unit conversion.

**Table 3:** Exposure factors used in this study

Categories	Age groups/scenarios	Value	Source
Body weight (kg)	0–9	13.21	Korean Exposure Factors Handbook for Children (MoE (Ministry of Environment) 2019a,b)
	10–18	54.53	Korean Exposure Factors Handbook for Children (MoE (Ministry of Environment) 2019a,b)
	19–65	65.30	Korean Exposure Factors Handbook (MoE (Ministry of Environment) 2019a,b)
	65–82	58.85	Korean Exposure Factors Handbook (MoE (Ministry of Environment) 2019a,b)
Inhalation rate (m^3^/day)	0–9	10.27	Korean Exposure Factors Handbook for Children (MoE (Ministry of Environment) 2019a,b)
	10–18	14.03	Korean Exposure Factors Handbook for Children (MoE (Ministry of Environment) 2019a,b)
	19–65	14.61	Korean Exposure Factors Handbook (MoE (Ministry of Environment) 2019a,b)
	65–82	14.60	Korean Exposure Factors Handbook (MoE (Ministry of Environment) 2019a,b)
Soil intake rate (mg/day)	0–9	35	Korean Exposure Factors Handbook for Children (MoE (Ministry of Environment) 2019a,b)
	10–18	10	US EPA Exposure Factors Handbook–Soil and Dust Ingestion (2017)
	19–65	10	US EPA Exposure Factors Handbook–Soil and Dust Ingestion (2017)
	65–82	10	US EPA Exposure Factors Handbook–Soil and Dust Ingestion (2017)
Outdoor exposure time (day)	0–9	0.029	Korean Exposure Factors Handbook for Children (MoE (Ministry of Environment) 2019a,b)
	10–18	0.028	Korean Exposure Factors Handbook for Children (MoE 2019)
	19–65	0.087	Korean Exposure Factors Handbook (MoE (Ministry of Environment) 2019a,b)
	65–	0.101	Korean Exposure Factors Handbook (MoE (Ministry of Environment) 2019a,b)
Indoor exposure time (day)	0–9	0.971	Korean Exposure Factors Handbook for Children (MoE (Ministry of Environment) 2019a,b)
	10–18	0.972	Korean Exposure Factors Handbook for Children (MoE (Ministry of Environment) 2019a,b)
	19–65	0.913	Korean Exposure Factors Handbook (MoE (Ministry of Environment) 2019a,b)
	65–	0.899	Korean Exposure Factors Handbook (MoE (Ministry of Environment) 2019a,b)
Exposure duration (day)	Virtual scenario	21	This study
	Real scenario	1	This study
Average time (day)	Virtual scenario	21	This study
	Real scenario	1	This study

The subjects for exposure assessment were divided by age into the following groups: 0–9 years, 10–18 years, 19–64 years, and > 65 years. Exposure factors, referenced from the Korean Exposure Factor Handbook (MoE (Ministry of Environment), 2019a,b) and the Korean Child Exposure Factor Handbook by the Ministry of Environment (MoE (Ministry of Environment) 2019a,b), were applied to these age groups. In addition, the amount of soil ingested by adults (data not available from the MoE) was referenced from data obtained from the US EPA (US EPA, 2017) (Table 4).

**Table 4:** AEGL of acrylic acid

Class	Exposure time				
	10 min	30 min	60 min	4 h	8 h
AEGL 1	1.5	1.5	1.5	1.5	1.5
AEGL 2	68	68	46	21	14
AEGL 3	480	260	180	85	58

It was assumed that the exposed subjects performed normal activities without much change after the chemical accident. Therefore, the inhalation exposure concentration was used. It depended on the exposure pathway, outdoor exposure concentration, and indoor exposure concentration. The outdoor exposure concentration was calculated using a DMEM. Considering that the leaked acrylic acid also moved indoors, the indoor exposure concentration was calculated using the indoor concentration prediction model (Eq. 1) developed by Park et al. (2018). Finally, the average daily dose (ADD) was determined using the calculated indoor and outdoor exposure concentrations (Eq. 2):

1 $${C}_{\mathrm{indoor}}=0.33{C}_{\mathrm{outdoor}}$$

where${C}_{\mathrm{indoor}}$: indoor toxic pollutant concentration (mg/m3).${C}_{\mathrm{outdoor}}$: outdoor toxic pollutant concentration (mg/m3)

2 $$\mathrm{ADD}(\mathrm{mg}/\mathrm{kg}/\mathrm{day})=\frac{\sum_{n=1}^{\mathrm{CLT}}\left({C}_{n \mathrm{outdoor}}\times \mathrm{IHR}\times \mathrm{OET}\right)+({C}_{n\mathrm{ indoor}}\times \mathrm{IHR}\times \mathrm{IET})}{\mathrm{BW}\left(\mathrm{kg}\right)\times \mathrm{AT}(\mathrm{day})}$$

where$\mathrm{CLT}$: period until the extinction of the target chemical in the environment (day).${C}_{n\mathrm{ outdoor}}$: concentration of the outdoor target chemical n days after accident occurrence (mg/m3).$\mathrm{IHR}$: inhalation rate (m3/day).$\mathrm{OET}$: outdoor exposure time (day).${C}_{n \mathrm{indoor}}$: concentration of the indoor target chemical n days after accident occurrence (mg/m3).$\mathrm{IET}$: indoor exposure time (day).$\mathrm{BW}$: body weight (kg).$\mathrm{AT}$: average exposure time (day).

Regarding ingestion exposure in the soil, the ADD calculation assumed complete acrylic acid absorption during ingestion (Eq. 3):

3 $$\mathrm{ADD}\left(\mathrm{mg}/\mathrm{kg}/\mathrm{day}\right)=\frac{\sum_{n=1}^{\mathrm{CLT}}({C}_{n \mathrm{soil}}\times {\mathrm{ITR}}_{\mathrm{Soil}})}{\mathrm{BW}(\mathrm{kg})\times \mathrm{AT}(\mathrm{day})}$$

where$\mathrm{CLT}$: period until the extinction of the target chemical in the environment (day).${C}_{n \mathrm{soil}}$: concentration of the target chemical in soil n days after accident occurrence (mg/kg).${\mathrm{ITR}}_{\mathrm{Soil}}$: soil intake rate (kg/day).$\mathrm{BW}$: body weight (kg).$\mathrm{AT}$: average exposure time (day).

Finally, the risk of a noncarcinogenic toxic substance was calculated using the HQ based on the reference dose and ADD from the exposure assessment (Eq. 4). RfC was changed to the same unit as RfD and used for the HQ calculation. When the calculated HQ exceeded 1, a health risk due to acrylic acid exposure was considered likely.

4 $$\mathrm{HQ}=\frac{\mathrm{ADD}(\mathrm{mg}/\mathrm{kg}/\mathrm{day})}{\mathrm{RfD}(\mathrm{mg}/\mathrm{kg}/\mathrm{day})}$$

where$\mathrm{HQ}$: hazard quotient.$\mathrm{ADD}$: average daily dose (mg/(kg·day)).$\mathrm{RfD}$: reference dose (mg/(kg·day)).

#### Acute exposure

To perform an acute health risk assessment, we monitored ordinary citizens’ exposure to air during a chemical leak or disaster using the Acute Exposure Guideline Level (AEGL) developed by the National Advisory Committee (NAC) under the US EPA (Table 5). Hourly assessments of the exposure concentration of acrylic acid in the air were calculated using the DMEM. The assessment ended when no part of the assessed area exceeded the AEGL. The exposure concentration in the air and hourly levels for AEGL-2 were compared to determine Hazard_acute_ AEGL-2 predicts a concentration in the air whereby the general population may experience serious and sustained adverse effects or inability to evacuate (NRC, 2001).

**Table 5:** Chronic risk assessment of inhalation exposure results (air)

Scenario	Ages	Exposure duration	Hazard quotient										
			Max	Mean	Percentile (%)								
					10	20	30	40	50	60	70	80	90
Virtual scenario	0–9	21 days	2176.23	10.71	4.41 × 10^−6^	2.28 × 10^−5^	7.32 × 10^−5^	1.32 × 10^−4^	2.61 × 10^−4^	6.98 × 10^−4^	2.06 × 10^−3^	4.00 × 10^−3^	8.73 × 10^−1^
	10–18		720.23	3.54	1.46 × 10^−6^	7.56 × 10^−6^	2.42 × 10^−5^	4.38 × 10^−5^	8.63 × 10^−5^	2.31 × 10^−4^	6.82 × 10^−4^	1.33 × 10^−3^	2.89 × 10^−1^
	19–65		696.22	3.43	1.41 × 10^−6^	7.30 × 10^−6^	2.34 × 10^−5^	4.24 × 10^−5^	8.34 × 10^−5^	2.23 × 10^−4^	6.59 × 10^−4^	1.28 × 10^−3^	2.79 × 10^−1^
	65 <		791.21	3.89	1.60 × 10^−6^	8.30 × 10^−6^	2.66 × 10^−5^	4.82 × 10^−5^	9.48 × 10^−5^	2.54 × 10^−4^	7.49 × 10^−4^	1.46 × 10^−3^	3.18 × 10^−1^
Actual accident	0 ~ 9	1 day	42.73	6.09 × 10^−2^	1.70 × 10^−2^	4.97 × 10^−14^	1.83 × 10^−9^	9.08 × 10^−7^	4.31 × 10^−5^	6.84 × 10^−4^	1.82 × 10^−3^	5.46 × 10^−3^	2.12 × 10^−2^
	10–18		14.14	2.02 × 10^−2^	5.64 × 10^−21^	1.65 × 10^−14^	3.06 × 10^−10^	3.00 × 10^−7^	1.43 × 10^−5^	2.26 × 10^−4^	6.04 × 10^−4^	1.81 × 10^−3^	7.00 × 10^−3^
	19–65		13.67	1.95 × 10^−2^	5.45 × 10^−21^	1.59 × 10^−14^	5.88 × 10^−10^	2.91 × 10^−7^	1.38 × 10^−5^	2.19 × 10^−4^	5.83 × 10^−4^	1.75 × 10^−3^	6.77 × 10^−3^
	65 <		15.54	2.22 × 10^−2^	6.19 × 10^−21^	1.81 × 10^−14^	6.68 × 10^−10^	3.31 × 10^−7^	1.57 × 10^−5^	2.49 × 10^−4^	6.63 × 10^−4^	1.98 × 10^−3^	7.69 × 10^−3^

Regarding Hazard_acute_, the value converting the AEGL represented in ppm, which serves as the threshold for acute exposure, into mg/m^3^ was compared; when the exposure concentration exceeded the threshold, a health risk arising from acrylic acid in the chemical accident was considered to occur (Eq. 5):

5 $${\mathrm{Risk}}_{\mathrm{acute}}=\frac{{C}_{n \mathrm{Air}}(\mathrm{mg}/{\mathrm{m}}^{3})}{\mathrm{AEGL}(\mathrm{mg}/{\mathrm{m}}^{3})}$$

where${\mathrm{Risk}}_{\mathrm{acute}}$: risk of acute.${C}_{n Air}$: hazardous pollutant concentration in the air over time after the accident (mg/m3).AEGL: AEGL-2 for hazardous pollutant (1 h) (mg/m3).

#### Risk map

To represent the risk assessment results in a more understandable and intuitive manner, the open-source Geographic Information System (GIS) program Quantum Geographic Information System (QGIS) V.3.10.10 was used to produce the risk map. QGIS was used to project a map with a 1:52,000 ratio scale onto the National Geographic Information Institute (NGII) topographical map. Similar to the DMEM, the risk map had a 0.1 km × 0.1 km grid in a 15 km × 12 km area. Red color represented the HQ and Hazard_acute_ > 1 as determined from the risk assessment, orange indicated 0.5–1, yellow indicated 0.1–0.5, light green indicated 0.1–0.05, and green indicated 0–0.05.

## Results

### Identifying the clearance time point for acrylic acid in the environment

#### Acrylic acid clearance time point in the hypothetical accident

In the hypothetical scenario involving 40 tons of acrylic acid leakage, the DMEM showed that the exposure concentration went below 0.01 ppb 21 days after the chemical accident. Therefore, the clearance time point of acrylic acid leakage in the hypothetical accident was determined to be 21 days, and this was used as the exposure period to conduct the risk assessment on chronic exposure.

#### Acrylic acid clearance time point in the actual accident

In the actual accident involving 1 ton of acrylic acid leakage, the DMEM showed that the exposure concentration went below 0.01 ppb 1 day after the chemical accident. The clearance time point of acrylic acid leakage in the actual accident was determined to be one day, and this was used as the exposure period to conduct the risk assessment on acute exposure.

### Chronic risk assessment results

#### Noncarcinogenic inhalation exposure (air)

**Fig. 1:**
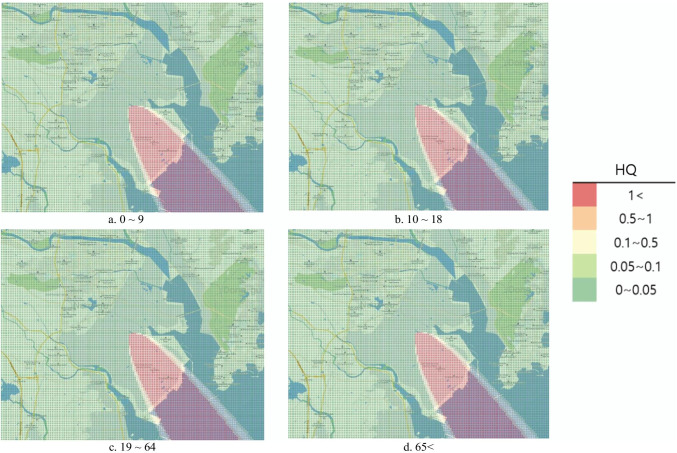
Hazard quotient of inhalation exposure risk map by age (virtual scenario)

Under the hypothetical accident scenario, the chronic HRA conducted on inhalation exposure in the air showed that the average HQ exceeded 1 across all age groups. Therefore, a health risk for local accidents in the affected area was considered likely (Table 6). In addition, the risk map representing the results for the hypothetical scenario showed the highest HQ southeast of the accident location across all age groups (Fig. 1).

**Table 6:** Chronic risk assessment of intake exposure results (soil)

Scenario	Ages	Exposure duration	Hazard quotient										
			Max	Mean	Percentile (%)								
					10	20	30	40	50	60	70	80	90
Virtual scenario	0–9	21 days	6.92 × 10^−5^	1.71 × 10^−7^	5.64 × 10^−15^	3.27 × 10^−14^	6.99 × 10^−14^	1.92 × 10^−13^	9.62 × 10^−13^	5.82 × 10^−12^	3.00 × 10^−11^	7.52 × 10^−11^	1.22 × 10^−8^
	10–18		4.79 × 10^−6^	1.18 × 10^−8^	3.91 × 10^−16^	2.26 × 10^−15^	4.84 × 10^−15^	1.33 × 10^−14^	6.66 × 10^−14^	4.03 × 10^−13^	2.08 × 10^−12^	5.21 × 10^−12^	8.41 × 10^−10^
	19–65		4.00 × 10^−6^	9.89 × 10^−9^	3.26 × 10^−16^	1.89 × 10^−15^	4.04 × 10^−15^	1.11 × 10^−14^	5.56 × 10^−14^	3.37 × 10^−13^	1.74 × 10^−12^	4.35 × 10^−12^	7.03 × 10^−10^
	65 <		4.44 × 10^−6^	1.10 × 10^−8^	3.62 × 10^−16^	2.09 × 10^−15^	4.48 × 10^−15^	1.23 × 10^−14^	6.17 × 10^−14^	3.73 × 10^−13^	1.93 × 10^−12^	4.82 × 10^−12^	7.80 × 10^−10^
Actual accident	0–9	1 day	8.05 × 10^−7^	1.68 × 10^−9^	4.97 × 10^−31^	2.19 × 10^−24^	1.74 × 10^−19^	1.91 × 10^−16^	1.47 × 10^−14^	5.10 × 10^−13^	6.49 × 10^−11^	2.35 × 10^−10^	1.78 × 10^−9^
	10–18		5.57 × 10^−8^	1.17 × 10^−10^	3.44 × 10^−32^	1.51 × 10^−25^	1.20 × 10^−20^	1.32 × 10^−17^	1.02 × 10^−15^	3.53 × 10^−14^	4.49 × 10^−12^	1.63 × 10^−11^	1.23 × 10^−10^
	19–65		4.65 × 10^−8^	9.74 × 10^−11^	2.87 × 10^−32^	1.26 × 10^−25^	1.00 × 10^−20^	1.11 × 10^−17^	8.51 × 10^−16^	2.95 × 10^−14^	3.75 × 10^−12^	1.36 × 10^−11^	1.03 × 10^−10^
	65 <		5.16 × 10^−8^	1.08 × 10^−10^	3.19 × 10^−32^	1.40 × 10^−25^	1.11 × 10^−20^	1.23 × 10^−17^	9.45 × 10^−16^	3.27 × 10^−14^	4.17 × 10^−12^	1.51 × 10^−11^	1.14 × 10^−10^

**Fig. 2:**
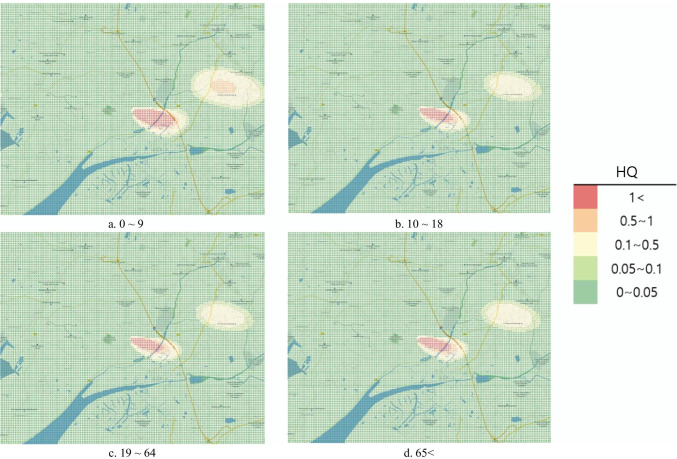
Hazard quotient of inhalation exposure risk map by age (actual accident)

Under the actual accident, the chronic HRA conducted on inhalation exposure in the air showed an average HQ of 6.29 × 10^−2^ in those aged 0–9, 2.22 × 10^−2^ in those aged 65 and older, 2.02 × 10^−2^ in those aged 10–18 years, and 1.95 × 10^−2^ in those aged 19–64 years. As the HQ did not exceed 1 in any age group, a health risk for local residents in the affected area was unlikely. However, the HQ exceeded 1 in regions within the affected area; therefore, the health risk was considered likely to occur locally (Table 6). The risk map representing the results of the actual accident shows the risk at the center and northeast of the accident location, and the highest HQ was detected from the center to the southeast (Fig. 2). Age group comparisons on the risk map showed the widest area with the HQ exceeding 1 in those aged 0–9 years.

#### Noncarcinogenic ingestion exposure (soil)

**Fig. 3:**
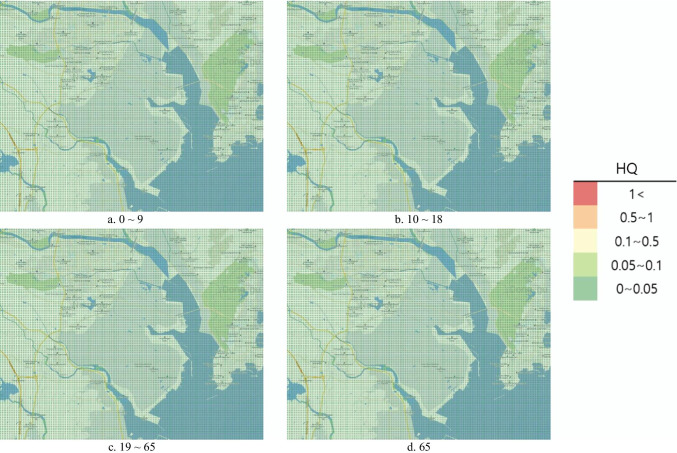
Hazard quotient of intake exposure risk map by age (virtual scenario)

Under the hypothetical accident scenario, the chronic HRA conducted on ingestion exposure in the air showed an average HQ of 1.71 × 10^−7^ in those aged 0–9 years, 1.18 × 10^−8^ in those aged 10–18 years, 1.10 × 10^−8^ in those aged > 65 years, and 9.89 × 10^−9^ in those aged 19–64 years (Table 6). The HQ did not exceed 1 in any age group, and the health risk for local residents due to acrylic acid ingestion exposure in the soil was considered unlikely (Table 7). The risk map representing the results under the hypothetical scenario did not exceed 1 across the area, which was green (Fig. 3).

**Table 7:** Acute risk assessment results

Scenario	Exposure time	Risk_acute_			
		Mean	Min	Mid	Max
Virtual scenario	1 h	4.49 × 10^−4^	0	0	4.25
	2 h	4.43 × 10^−2^	0	0	7.28
Actual accident	1 h	2.87 × 10^−9^	0	0	6.41 × 10^−5^
	2 h	7.03 × 10^−6^	0	0	7.89 × 10^−3^

**Fig. 4:**
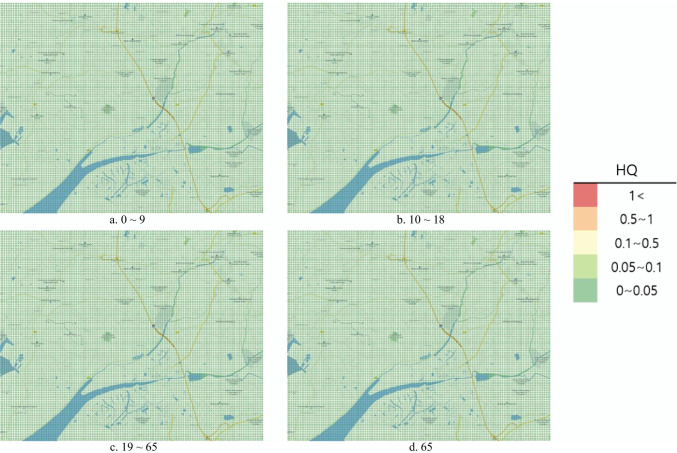
Hazard quotient of intake exposure risk map by age (actual accident)

The chronic HRA conducted to analyze ingestion exposure during the actual accident revealed the average HQ to be 1.68 × 10^−9^ in those aged 0–9 years, 1.17 × 10^−10^ in those aged 10^–18^ years, 1.08 × 10^−10^ for those aged > 65 years, and 9.74 × 10^−11^ for those aged 19–64 years. The HQ did not exceed 1 in any age group, and the health risk for local residents due to acrylic acid ingestion exposure in the soil was considered unlikely (Table 7). The risk map representing the results under the actual accident did not exceed 1 across the area, which was green (Fig. 4).

### Acute risk assessment results

**Fig. 5:**
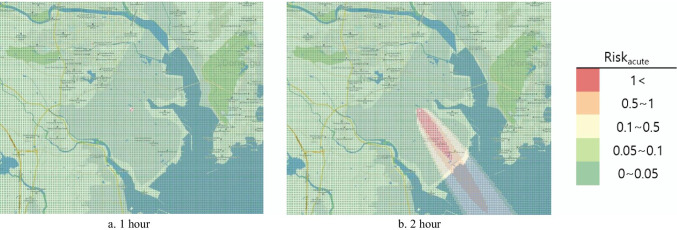
Risk_acute_ risk map by time (virtual scenario)

The acute HRA under the hypothetical accident scenario found that the risk likely persisted for up to 2 h after the accident, and it was unlikely to be present after 3 h, as the Hazard_acute_ did not exceed 1 (Table 8). Furthermore, the risk map representing the results under the hypothetical scenario showed that the Hazard_acute_ was most widely distributed 2 h after the accident, and it vanished rapidly after 3 h, and all the areas turned green (Fig. 5).

**Fig. 6:**
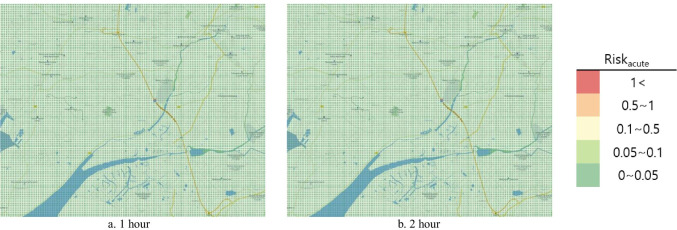
Risk_acute_ risk map by time (actual accident)

The acute HRA found that the risk was unlikely in the actual accident case, as the Hazard_acute_ did not exceed 1 after the accident (Table 8). In addition, the risk map representing the results under the hypothetical scenario showed that all the areas were green after the accident, indicating no risk (Fig. 6).

## Discussion

This study used the DMEM to assess chemical exposure and the likelihood of acute and chronic health risks, among local residents, following a hazardous chemical accident. A risk map was prepared so that the health impact could be understood intuitively.

The exposure factors used to conduct the HRA in this study should have reflected activities performed over time, such as an evacuation and the return-to-normal after the chemical accident. However, there has been no standardized study on such activities. Therefore, general public activities reported by the National Institute of Environmental Research (2019) were included in this study. Consequently, a decrease in exposure due to evacuation after the accident was not reflected, which may have exaggerated the risk assessment results. However, considering the purpose of this study, which is to provide a reference for establishing response and post-accident management options to protect local residents during a chemical accident, preparing these options based on the results of the exposure and HRA by considering the maximum potential exposure is essential. Future studies should also consider the maximum potential exposure during analysis.

A literature review was conducted on previous studies in Korea investigating hazardous chemical background concentrations to identify the clearance time point of acrylic acid in the environment. It was found that there were not enough data regarding the background concentrations of acrylic acid. Hence, at the author’s discretion, the endpoint concentration was assumed to be 0.01 ppb, the detection limit of most measuring instruments. In the future, the background concentrations of substances requiring preparation for accidents under the Chemical Substances Control Act, including acrylic acid, should be designated and studies conducted to determine their endpoint concentration based on the DMEM. These steps will allow the clearance time point of the chemicals in the environment to be identified accurately.

The risk posed to those aged 0–9 years was the highest among all groups, which can be attributable to a higher inhalation rate per kilogram of body weight than in adults. This finding suggests that the accident response and post-accident management options in a chemical accident must prioritize vulnerable groups, including children.

The chronic HRA of soil ingestion exposure revealed no health impact on local residents across all age groups under any scenario. Unlike children, soil ingestion exposure seemed to rarely occur in adults as they were less likely to touch the soil. Although the exposure to soil ingestion was not high in children, they were more likely to touch the soil due to a lack of artificial green spaces and the use of urethane to construct children’s playgrounds in Korea (National Institute of Environmental Research, 2019).

**Fig. 7:**
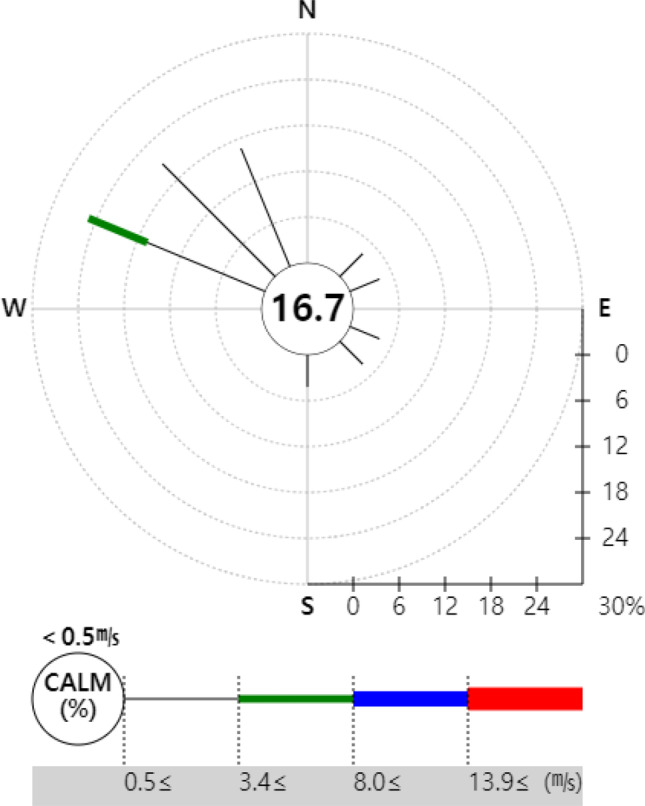
Wind rose of Ulsan-Si Metropolitan City during the Risk assessment period (KMA 2020)

The acute risk assessment showed that under the hypothetical scenario, local residents were exposed to high concentrations of acrylic acid in large numbers shortly after the accident, indicating that an accident response to acute exposure must be implemented rapidly. However, no acute health impact was found in the actual accident case. This finding does not mean that there was no health impact. Instead, it seems to suggest that the exposure was underestimated due to the advection and diffusion of the hazardous chemical as it leaked continuously for 1 h in the air, as the DMEM assessed exposure on an hourly basis. Thus, it would be better to assess the resolution of the predicted exposure concentration in the model on a minute-by-minute basis. Furthermore, the minimum unit of time in the AEGL, used as the threshold for acute exposure in this study, was 10 min. Therefore, calculation on a minute-by-minute basis would make it possible to build a more systematic assessment of health impact and accident response for acute exposure (Fig. 7).

**Fig. 8:**
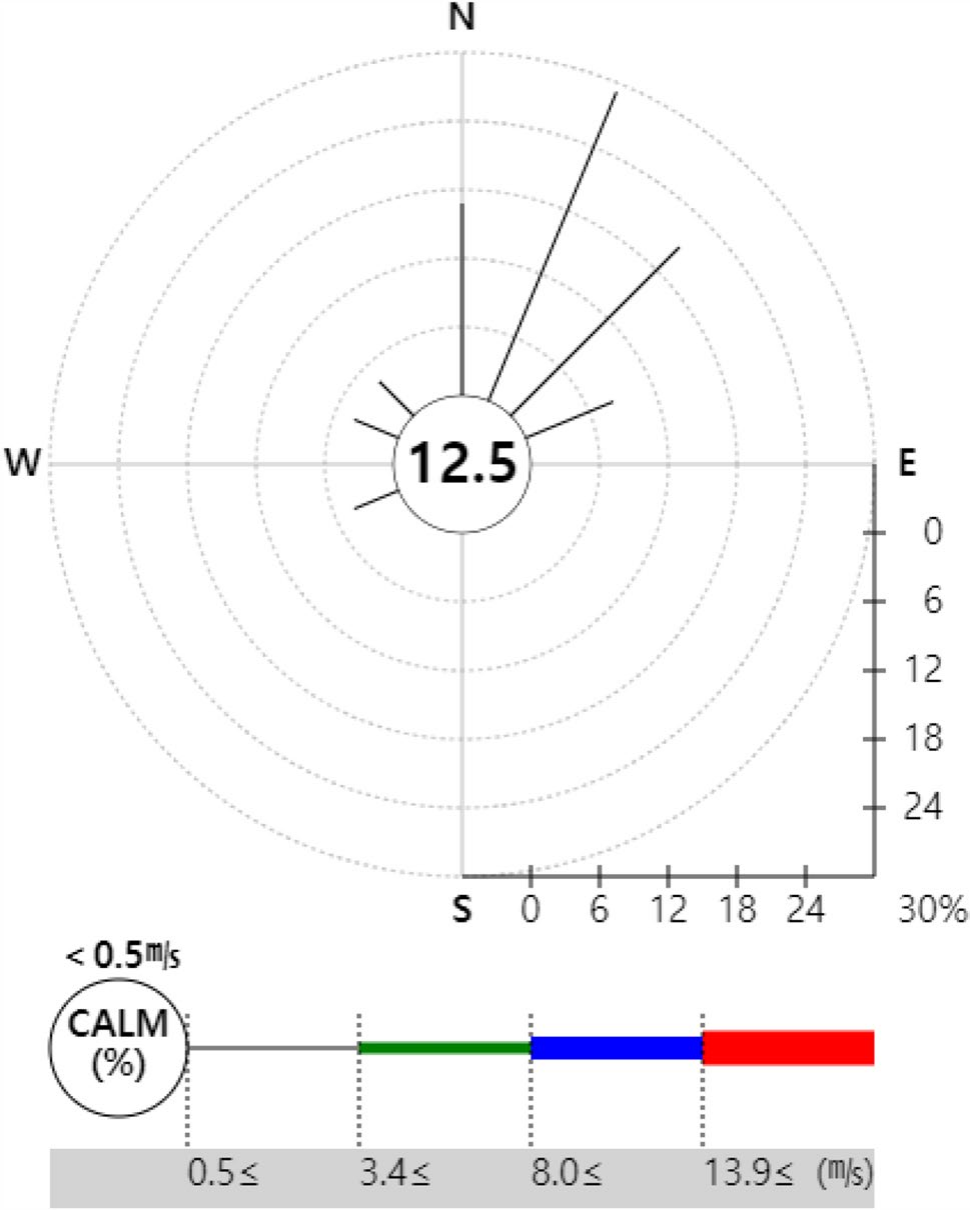
Wind rose of Hwasung-Si Metropolitan City during the Risk assessment period (KMA 2020)

The risk map representing the results of the chronic HRA due to inhalation exposure in the air showed that the risk was distributed southeast of the accident location under the hypothetical scenario, while the wind based on weather conditions in Ulsan showed that it was consistent with the actual wind direction in the city. The wind mostly blew in a northeasterly direction in Hwaseong during the actual accident case, but the wind speed in Hwaseong was lower than that in Ulsan; consequently, the chemical moved northeast or southwest, not center or northeast, as it was affected by the wind from other directions (Fig. 8). This implies that not only the wind direction but also the wind speed is greatly involved in the movement of a chemical during a leak accident; thus, in the future, it would be necessary to consider the wind speed in addition to the main wind direction to predict the movement of a chemical in an accident.

This study identified the concentration of acrylic acid in a chemical accident by using a DMEM that considered a hazardous chemical's behaviors in the environment and its changing concentrations over time. In addition, the study found the clearance time point in the environment, conducted an appropriate health risk assessment, and reflected the characteristics of exposure in a chemical accident. Furthermore, we also compared the results of the HRA between the hypothetical scenario and the actual accident case and provided a reference for developing HRA techniques for chemical accidents.

## Conclusions

This study focused on acrylic acid, one of the substances requiring preparation for accidents, in a hypothetical accident scenario and an actual accident case using the DMEM developed by our team and compared the results of acrylic acid exposure and HRA of local residents in the area affected by the accident. The following conclusions were drawn based on the results of the study.

According to the chronic HRA conducted on the inhalation exposure of acrylic acid remaining in the air, the average HQ suggested that a health risk was likely for local residents in the accident-affected area under the hypothetical scenario. Under the actual accident case, the average HQ exceeded 1 in some parts of the area, indicating that health risks may occur locally.

Hazard_acute_ exceeded 1 just 2 h after the accident under the hypothetical scenario for the acute HRA, suggesting that a health risk was likely for local residents in the area affected by the accident. The risk map representing these results showed the widest Hazard_acute_ 2 h after the accident.

The results of this study are expected to be useful in developing chemical accident response and post-accident management planning, as it suggests a methodology that can assess changing concentrations of a hazardous chemical leaked during a chemical accident based on time, place, movement, advection, diffusion, and clearance in different environmental media, as well as the health risk posed by the exposure of local residents to the chemical.

## Data Availability

Not applicable.

## References

[CR1] Chemical Information System, *NICS (National Institute of Chemical Safety)* (2020) https://icis.me.go.kr.

[CR2] Hong HJ, Park SH, Lim HB, Lee CM (2020). Development on health risk assessment method for multimedia exposure of hazardous chemical by chemical accident. Int J Environ Res Public Health.

[CR3] Jo GY, Lee IM, Hwang YW, Moon JY (2017). A study on the simulation of damage distance for toxic substances leakage. Korea Academy Industrial Cooperation Society.

[CR4] Kim EB, Oh JY, Lee TW, Oh WK, Kim HJ, Lim DY (2020). A study on the development of GIS-based complex simulation prototype for reducing the damage of chemical accidents. Korean Journal of Remote Sensing.

[CR5] Kim SY, Cho CH, Lee EK (2017). Studies on the chemical accidents of Korea by the statistics and case review. Korean Journal of Hazardous Materials.

[CR6] KMA (Korea Meteorological Administration) (2020) Meteorological Data Portal. https://data.kma.go.kr/cmmn/main.do. Accessed 21 Feb 2021

[CR7] Lee CH (2013). Problems and prevention measures of recent chemical accidents.

[CR8] Park SH, Lim HB, Hong HJ, Yoon DK et al (2020) Health risk assessment for multimedia exposure of formaldehyde emitted by chemical accident. Environmental Science and Pollution Research 28(8), 9712-9722. 10.1007/s11356-020-11403-w 33151492

[CR9] NIER(National Institute of Environmental Research) (2019). Korean exposure factors handbook.

[CR10] NIER(National Institute of Environmental Research) (2019). Korean exposure factors handbook for children. 2019

[CR11] NRC (National Research Council) (2001). Standing operating procedures for developing acute exposure guideline levels for hazardous chemicals. 10.17226/10122 25057561

[CR12] Park SH, Yoon DK, Park TH, Hong HJ, Lee ES (2018). A study on the prediction of hazardous substance concentration in the indoor space by indoor inflow of chemical accident material. Proceedings of the Korean Environmental Science Society.

[CR13] Park SH, Lim HB, Hong HJ, Kim HS, Yoon DK (2020). Health risk assessment for multimedia exposure of formaldehyde emitted by chemical accident. Environ Sci Pollut Res.

[CR14] PubChem. National Institutes of Health. https://pubchem.ncbi.nlm.nih.gov/compound/6581#section=Information-Sources. Accessed 5 Feb 2021

[CR15] Quantum geographic information system. https://www.qgis.org/en/site/. Accessed 5 Feb 2021

[CR16] US EPA (United States Environmental Protection Agency) (2017) Update for Chapter 5 of the exposure factors handbook-soil and dust ingestion-, EPA/600/R-17/384F

[CR17] US EPA (United States Environmental Protection Agency) (2018) Compiled acute exposure guideline values (AEGLs). https://www.epa.gov/sites/default/files/2018-08/documents/compiled_aegls_update_27jul2018.pdf. Accessed 5 Feb 2021

[CR18] US EPA (United State Environmental Protection Agency) integrated risk information system (IRIS). https://www.epa.gov/iris. Accessed 5 Feb 2021

